# Caregiver Report of Executive Functioning in Adolescent Females With Anorexia Nervosa or Autism Spectrum Disorder

**DOI:** 10.3389/fpsyg.2020.586264

**Published:** 2021-02-09

**Authors:** C. Alix Timko, John D. Herrington, Anushua Bhattacharya, Emily S. Kuschner, Benjamin E. Yerys

**Affiliations:** ^1^Department of Child and Adolescent Psychiatry and Behavioral Sciences, Children’s Hospital of Philadelphia, Philadelphia, Pennsylvania; ^2^Department of Psychiatry, Perelman School of Medicine, University of Pennsylvania, Philadelphian, Pennsylvania

**Keywords:** anorexia nervosa, autism spectrum, executive functioning, set shifting, female

## Abstract

Current literature suggesting a shared endophenotype between individuals with anorexia nervosa (AN) and autism spectrum disorder (ASD) related to executive functioning (EF) has several limitations: performance-based instead of ecologically valid measures of set-shifting are used, lack of comparisons between same-sex groups, and reliance on adult samples only. This was the first study directly comparing female youth with ASD to female youth with AN using an ecologically valid measure of EF. A secondary data analysis combined caregiver-reported EF on the Behavior Rating Inventory of Executive Functioning (BRIEF) for 22 female adolescent youth with AN and 29 female adolescent youth with ASD. EF in each group was compared to population norms, and EF was compared between groups. Compared to population norms, adolescents with AN had elevated scores on shift, initiate, and emotional control scales, and adolescents with ASD had elevated scores on all scales of the BRIEF and were more likely to have scores in the clinical range. There were significant differences between groups on all but three scales. The cognitive profiles and clinical scores of AN females were not comparable to those of ASD females. The findings reveal a clear clinical impairment in females with ASD but not in females with AN. The results do not support the hypothesis of similar real-world EF profiles between these groups. The results encourage further exploration into the similarities and distinctions between these two disorders.

## Introduction

An emerging body of literature posits a putative link between anorexia nervosa (AN) and autism spectrum disorder (ASD), specifically that ASD and AN share a genetic risk and/or endophenotypes that result in high degrees of comorbidity (Huke et al., 2013; Mandy and Tchanturia, 2015). Both disorders are biologically based and can be characterized by cognitive inflexibility and social communication difficulties accompanied by repetitive restricted behaviors and interests. Overlapping characteristics between AN and ASD have been observed clinically (Kerbeshian and Burd, 2009; Bandini et al., 2010) and through self-report (Westwood et al., 2017; Bitsika and Sharpley, 2018). Extant research investigating the overlap between ASD and AN has focused on either socio-emotional functioning or executive functioning (EF). The former work concentrated primarily on identifying difficulties in emotional theory of mind (Leppanen et al., 2018; Sedgewick et al., 2019) or autistic traits in individuals with AN; (Oldershaw et al., 2010; Kerr-Gaffney et al., 2019) research in the area of EF highlights shared inefficiencies in set-shifting (Holliday et al., 2005; Wong et al., 2006). Also referred to as cognitive flexibility, set-shifting describes an individual’s ability to easily switch between tasks, change mindsets, rapidly adapt to new situations, shift attention to different features of objects or situations, and respond to new contingencies in the environment. There are documented impairments in set-shifting in individuals with ASD (Demetriou et al., 2017; Lai et al., 2017; Zhang et al., 2020) and evidence for set-shifting inefficiencies in adults with AN (Roberts et al., 2007; Hirst et al., 2017). The latter do not appear to be state-based, as they do not fully reverse with re-nourishment (Tchanturia et al., 2002; Danner et al., 2012).

Current publications exploring the hypothesis that set-shifting inefficiencies may be a trait shared by AN and ASD (Treasure, 2013) have a number of limitations: (1) comparisons are between collected data from one cohort compared to published results of another cohort, (2) the AN and ASD samples are not clearly defined and matched, (3) sex as a biological variable has not been controlled for, and (4) the average age of the participants in the published work is in the young adult range despite AN and ASD being disorders with an onset during adolescence and childhood, respectively. Adult samples are particularly problematic, as EF begins to develop in infancy (ASD symptoms begin to emerge in the second year of life) and continues to develop through adolescence (when AN typically emerges), with some skills reaching full maturity only in early adulthood. While difficulties in EF in individuals with ASD are well documented, (Sanders et al., 2008) it is difficult to know whether observed set-shifting inefficiencies in adults with AN are truly an endophenotype of the disorder, if they are an endophenotype for a chronic course, or if they are a scar of severe malnutrition that occurred during a key period of brain development. The question of whether or not inefficiencies in set-shifting are a result of illness in AN is particularly salient, as data on set-shifting inefficiencies in adolescents with AN are equivocal. A number of hypotheses have been set forth as to why we do not regularly observe inefficiencies in adolescents, including the reliance on performance-based measures of EF. Performance-based measures are typically administered under ideal testing conditions, are not necessarily reflective of real-world performance, and may not be as sensitive to inefficiencies in adolescents as they are in adults (Stedal and Dahlgren, 2015). When examining profiles of youth with ASD and AN, ecologically valid measures may provide a complimentary and slightly different picture than performance-based measures.

Although published literature compares women with AN to males with ASD, (Oldershaw et al., 2011) examining similarities or differences in set-shifting in the same sex sample is needed. Some research suggest that female youth with ASD have more difficulties in EF than males with ASD; (White et al., 2017) there are not enough published data to compare males and females with AN on EF. We assessed EF in female youth with AN and female youth with ASD using a rigorous, ecologically valid measure (Gioia et al., 2000) and hypothesized that both groups would have greater impairments in set-shifting than published norms. We further hypothesized that girls with AN would have clinical impairments (using published norms) in inhibition, shifting, and self-monitoring. Given the nature of this secondary data analysis, we wanted to be targeted in our *a priori* hypothesis. Thus, we focused on shifting, inhibition, and (for AN) monitoring, as it reflects an individual’s ability to understand their impact on others. The constructs assessed by these specific scales have been implicated in prior research as relevant for AN; furthermore, in the unity/diversity model (Friedman and Miyake, 2017), inhibition and shifting are noted as two of the three main constructs of EF (the third is updating). Finally, we planned exploratory analyses of subscales measuring EF impairments to test a secondary hypothesis that these inefficiencies would be positively correlated with eating disorder severity for the AN group (Yerys et al., 2009) and repetitive behaviors for the ASD group (Kenworthy et al., 2009).

## Materials and Methods

### Participants

The participants were pooled from previously completed studies conducted by the Center for Autism Research and the Eating Disorder Research Program at the Children’s Hospital of Philadelphia^1^. All procedures involving human patients were approved by the Institutional Review Board of the hospital. Written informed consent was obtained from the caregivers of all patients. Females (*n* = 29) in the ASD sample were drawn from four different studies. All met the DSM-IV criteria for either autism, Asperger’s syndrome disorder, or pervasive developmental disorder—not otherwise specified. At the time of data collection (2009–2015), we used the DSM-IV-TR and DSM-5 (when published) checklists for an ASD diagnosis (specifically autism, Asperger’s, or PDD-NOS). Expert clinicians trained to research reliability administered the Autism Diagnostic Observation Schedule (Lord et al., 2000) with revised algorithms, (Gotham et al., 2007) the Autism Diagnostic Interview-Revised, (Lord et al., 1994) and the Social Communication Questionnaire (Rutter et al., 2003).

Females (*n* = 22) in the eating disorder sample were drawn from two separate studies. Diagnosis was made *via* a clinical interview by eating disorder specialists and confirmed *via* the Eating Disorder Examination (Fairburn et al., 2014).

### Materials

All participants had at least one caregiver complete the caregiver version of the Behavior Rating Inventory of Executive Functioning (BRIEF) (Gioia et al., 2000). The BRIEF is an ecologically valid clinical tool for measuring EF across several domains in youth 5 to 18 years of age (Gioia et al., 2000). Its eight clinical scales and two indices are described in Table 1. The BRIEF is normed by age and sex on a T-scale (mean = 50, SD = 10), and scores are considered clinically elevated if they are 65 or higher. We used a caregiver report of EF, as valid self-reports can be challenging for children and adolescents with ASD (Kerns et al., 2015). Research indicates that parental figures can more broadly assess the executive function of their youth in real-life scenarios through the BRIEF (Kenworthy et al., 2008; Wallace et al., 2016). In order to gain a clearer picture of both groups’ scores on all indices, we also compared the percentage of adolescents who had elevated scores above the threshold of clinical concern on each subscale.

**TABLE 1:** Descriptions of the eight clinical subscales and two indices of the Behavior Rating Inventory of Executive Functioning.

	**Description**
**Clinical subscale**	
Inhibit	Ability to resist impulses and appropriately halt a behavior in a timely manner
Shift	Ability to freely switch attention or topic and to accept change
Emotional control	Ability to regulate one’s feelings appropriately
Initiate	Ability to start an activity on one’s own and develop one’s own opinions, ideas, and solutions
Working memory	Ability to recall information useful for carrying out a task and achieving goals
Plan/organized	Ability to prepare, develop, and bring to fruition goals and future events and to comprehend the main concepts of written or verbal work
Organization of materials	Ability to systematically arrange spaces (e.g., storage, bedrooms, lockers)
Monitor	Ability to evaluate one’s work and performance and to evaluate one’s behavior and its impact on others
**Index**	
Behavioral regulation index	A measure of how well a child controls his behavior and adapts to change in the environment
Metacognition index	An adolescent’s ability to control and organize his thoughts
Global executive functioning	Overall global score of executive functioning

For adolescents with ASD, IQ was determined by either the Differential Ability Scale (Elliot, 2007) or a Wechsler IQ instrument [e.g., Wechsler Abbreviated Scale of Intelligence-2 (WASI-II) (Wechsler and Hsiao-pin, 2011)]; for adolescents with an eating disorder, the two-scale version of the WASI-II was used to determine the IQ. In addition, we obtained the disorder-specific behavior for each group. For adolescents with ASD, we used the Repetitive Behavior Scale-Revised (RBS-R) (Bodfish et al., 2000) to broadly assess restricted and repetitive behaviors (RRBs), a core symptom domain of ASD. The RBS-R is a 43-item caregiver report; behaviors are rated on a 0 (“behavior does not occur”) to 3 scale (“behavior occurs and is a severe problem”). The dependent variable of interest is the overall score, which ranges from 0 to 129. For adolescents with an eating disorder, we included the body mass index (BMI) *z*-score from the time of assessment and the scores on the Eating Disorder Examination (EDE) (Fairburn et al., 2014). The EDE is a semi-structured interview that queries eating disorders and has four scales: restraint, eating concern, shape concern, and weight concern; when combined, the four scales create a global score. Given the *post hoc* analytic design of the present study, data on weight and eating disorder symptoms were not available for youth with ASD; data on RRBs were not available for youth with AN.

### Procedure

Experienced clinicians made the diagnoses for youth in both samples based on appropriate diagnostic interviews and in accordance with DSM-IV and DSM-5 criteria for the ASD and AN sample, respectively. Trained health professionals obtained written informed consent or assent by all participants and written informed consent from their parent/guardian. The BRIEF was administered as part of a larger behavioral battery in each of the respective studies.

### Data Analyses

In order to determine whether or not the average score in each group differed from population norms, we conducted a series of one-sample *t*-tests for each group separately. Although we were specifically interested in the shift, inhibit, and monitor scales, we conducted a multivariate analysis of covariance (MANCOVA) with group (ASD vs. AN) as the independent variable and age [as EF worsens with age in youth with ASD (Rosenthal et al., 2013)] and IQ as covariates. The dependent variables were the subscales of the BRIEF. The correlation matrix for the subscales of the BRIEF indicated that they were all at least moderately correlated; thus, it was appropriate to conduct a MANCOVA. We followed the MANCOVA with discriminate analysis to determine if there was a difference between groups in EF as a whole. We used regression analyses to examine the relationship between scores on the BRIEF with population-specific phenotypes.

As this was a secondary data analysis, we were not able to conduct an *a priori* power analysis. However, a *post hoc* power analysis informed by published data (Leung and Zakzanis, 2014) indicated that, for the ASD sample, an *N* of 4 would be needed to detect a group difference from the standardization mean of *T* = 50. In what we believe to be the only published report comparing caregiver report on the BRIEF for adolescents with AN (*N* = 24) and healthy controls (*N* = 37), adolescents with AN had significantly higher scores than healthy controls (*d* =.73) (McAnarney et al., 2011). Other published data using the BRIEF to assess set-shifting inefficiencies had comparable sample sizes of 20 (Stedal and Dahlgren, 2015) and 40 (Herbrich et al., 2019). Thus, the sample of adolescents with AN is comparable to published work and, based on prior data, should be sufficiently powered to find a difference from published norms.

## Results

Demographics (age, IQ, and ethnicity) of both samples are displayed in Table 2. For the ASD sample, the average score for RRBs was 19.43 (SD = 14.59, range 1–73). For the AN sample, the average BMI *z*-score was −1.44 (SD = 1.21), and BMI percentiles ranged from 0 to 89% (*M* = 18.10, SD = 22.03). The average global score on the eating disorder examination was 2.60 (SD = 2.09), with a range of 0–6. The average length of illness was 19.43 months (SD = 15.66), with a range of 2–59 months.

**TABLE 2:** Demographic description of the participants.

	**Anorexia nervosa (*n* = 22)**			**Autism spectrum disorder (*n* = 29)**		
	**M**	**SD**	**Range**	**M**	**SD**	**Range**
Age	14.81	2.33	10–18	12.30	2.25	10–17
IQ	105.84	13.95	83–142	96.3	24.69	42–145
**Ethnicity**						
Caucasian	90.9%			94%		
Hispanic	4.5%			3%		
African American	4.5%			3%		

### Comparison of ASD to AN Sample to Population Norms

**FIGURE 1:**
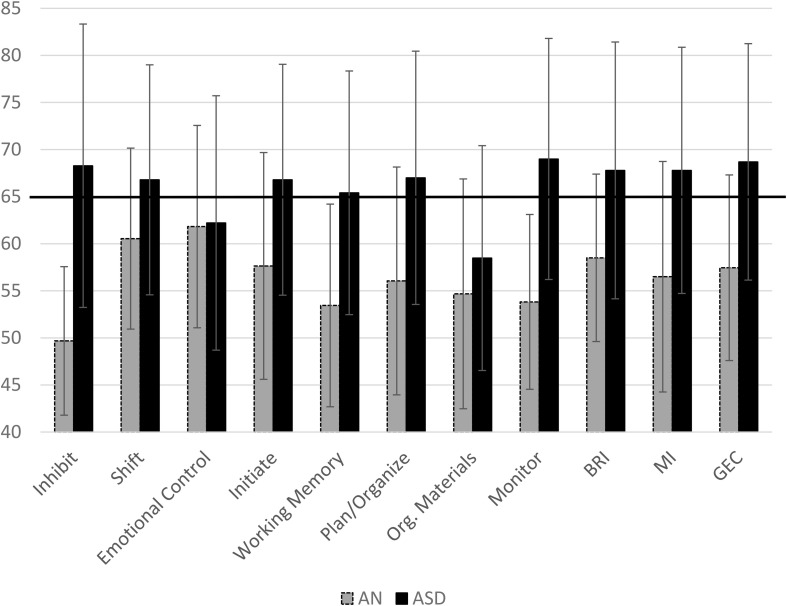
Mean scores for the AN and ASD group on each subscale of the BRIEF with Standard Deviation bars. Clinical cutoff is 65. Em. Cont.,Emotional Control; Work. Mem.,Working Memory; Plan/Org,Plan/Organization; Org. Materials, Organization of Materials; BRI, Behavioral Regulation Index; MI, Metacognition Index; GEC, Global Executive Composite. BRI, MI, and GEC are indices.

**FIGURE 2:**
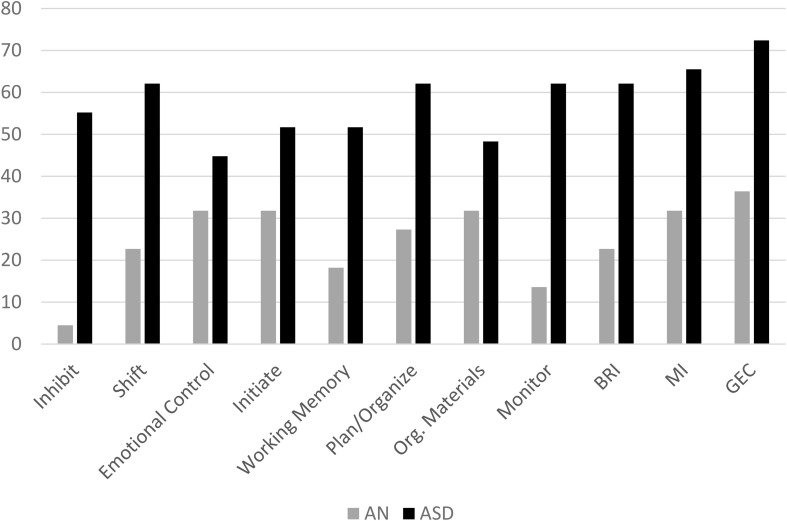
Percent of Adolescents scoring in the Clinical Range on Subscales of the BRIEF.Org. Materials, Organization of Materials; BRI, Behavioral Regulation Index; MI, Metacognition Index; GEC, Global Executive Composite. BRI, MI, and GEC are indices.

The mean *T*-scores for each group on all subscales of the BRIEF are displayed in Figure 1. Adolescents with AN had significantly higher scores on shift, initiate, plan/organize, and emotional control scales as well as on the metacognitive index, behavioral regulation index, and the global executive composite compared to population norms (see Table 3). Adolescents with ASD had higher scores than population norms on all subscales and all indices. A higher percentage of adolescents with ASD had scores in the clinical range than those with AN; in fact, less than 5% of adolescents with AN had elevated scores on the inhibit scale, 22.7% had elevated scales on the shift scale, and 13% had clinical scores on the monitor scale (Figure 2). Approximately 30% of adolescents with AN had elevated scores on the emotional control, initiate, and organization of materials scales, and 27% of adolescents with AN had elevated scores of the plan/organize scale. In contrast, over 50% of all adolescents with ASD had elevated scores on every scale, with the exception of the emotional control and organization of materials scales.

**TABLE 3:** Mean and standard deviation for adolescents with anorexia nervosa (AN, *N* = 22) and autism spectrum disorder (ASD, *N* = 29) and their comparison to population norms (*M* = 50).

	**Group**	***M***	**SD**	***t***	***P***	***d***	**95% confidence interval**
Inhibit	AN	49.68	7.888	–0.189	0.852	–0.040	−3.82–3.18
	ASD	68.28	15.043	6.543	0.000	1.215	12.55–24.00
Shift	AN	60.45	9.610	5.103	0.000	1.088	6.19–14.72
	ASD	66.79	12.211	7.406	0.000	1.375	12.15–21.44
Monitor	AN	53.82	9.282	1.929	0.067	0.411	−0.30–7.93
	ASD	69.00	12.798	7.995	0.000	1.485	14.13–23.87
Emotional Control	AN	61.82	10.747	5.158	0.000	1.100	7.05–16.58
	ASD	62.21	13.508	4.867	0.000	.904	7.07–17.34
Initiate	AN	57.64	12.042	2.974	0.007	0.634	2.30–12.98
	ASD	66.79	12.257	7.378	0.000	1.37	12.13–21.46
Working memory	AN	53.45	10.760	1.506	0.147	0.321	−1.32–8.23
	ASD	65.41	12.938	6.416	0.000	1.191	10.49–20.34
Plan/organize	AN	56.05	12.097	2.344	0.029	0.500	0.68–11.41
	ASD	67.00	13.448	6.807	0.000	1.264	11.88–22.12
Organization of materials	AN	54.68	12.198	1.800	0.086	0.384	−0.73–10.09
	ASD	58.48	11.942	3.825	0.001	0.710	3.94–13.03
Behavior regulation index	AN	58.50	8.890	4.485	0.000	0.956	4.56–12.44
	ASD	67.69	13.634	7.028	0.000	1.305	12.61–22.98
Metacognition index	AN	56.50	12.239	2.491	0.021	0.531	1.07–11.93
	ASD	67.79	13.070	7.331	0.000	1.361	12.82–22.76
Global executive composite	AN	57.45	9.855	3.548	0.002	0.756	3.09–11.82
	ASD	68.69	12.559	8.041	0.000	1.488	13.91–23.47

### Between-Group Comparisons

Age and IQ were entered as covariates in the original MANCOVA; however, only IQ was significant. We removed age from the model and reran the analysis. Given the small and unequal sample sizes, we ensured that we had equality of the covariance matrix [Box’s test: *F*(36, 5,017.690) = 1.158, *p* = 0.238] for the MANCOVA. Pillai’s trace was significant [0.622, *F*(8, 38) = 7.803, *p* < 0.001, *η_*p*_*^2^ = 0.622]. We conducted a *post hoc* discriminate analysis in order to examine whether or not EF (as a linear function of all subscales) would differentiate the groups. One factor explained 100% of the variance (canonical *R*^2^ = 0.804). Adolescents with ASD and adolescents with AN were significantly different in EF [*Λ* = 0.354, *χ*^2^(8) = 46.743, *p* < 0.001]. The majority (96.1%) of all participants were correctly classified by a linear combination of all scores on the BRIEF. Importantly, all adolescents with AN were correctly classified and two adolescents with ASD were incorrectly classified as belonging to the AN group.

### Relationship Between EF and Population Specific Phenotypes

To determine if shift was associated with RRBs in subjects with ASD, we conducted a hierarchical linear regression with age and IQ entered in the first step and scores on the shift entered on the second step. The mean was entered for any missing data. The first model was not significant (*p* = 0.26). Adding shift scores significantly increased the fit of the model *R*_Δ_^2^ = 0.271, *F*_Δ_ (1, 25) = 10.73, *p* = 0.003, which was significant [*R*^2^ = 0.369, *F*(3, 25) = 4.876, *p* = 0.008). Similar to Kenworthy et al. (2009), age (β = −0.167, *t* = −1.017, *p* = 0.32, 95% CI: −3.229, 1.095) and IQ (β = −0.085, *t* = −0.533, *p* = 0.599, 95% CI: −0.236, 0.139) did not predict RRBs, whereas shift scores did (β =.540, *t* = 3.276, *p* = 0.003, 95% CI: 0.235, 1.032).

For adolescents with AN, we conducted a similar (illness-relevant) analysis, with age and IQ entered in the first step, BMI z-score and length of illness in the second, and shift, inhibit, and monitor in the third. We used the global score of the EDE as the dependent variable. The mean was entered for any missing data. The model was not significant at any step, and the addition of variables at each stage did not significantly improve the fit of the model [final model: *R*^2^ = 0.288, *F*(7, 14) = 0.808, *p* = 0.595].

## Discussion

This was the first study directly comparing female youth with ASD to female youth with AN using an ecologically valid measure of EF. Prior research directly compared adult samples of AN and ASD on EF, confounding the effects of the AN phenotype with those of starvation alone (Courty et al., 2013). Furthermore, prior research used traditional neuropsychological tests of EF (e.g., Wisconsin Card Sorting Test, Stroop), which are usually administered in ideal circumstances and thus have poor to moderate ecological validity. To provide complementary information to performance-based measures previously presented in the literature, (Isquith et al., 2013) we used an ecologically valid measure that provided an assessment of the behavioral manifestation of difficulties in EF in these two populations. It is important to note that we used a caregiver report of their child’s EF. The caregiver report is frequently used with adolescents with ASD who may struggle with providing a self-report (Kerns et al., 2015) thus caregivers are more likely to give accurate reports.

We hypothesized that adolescent females with AN and ASD would have higher scores than population norms. Females with ASD in this sample had elevated scores on scales within the EF profile that are consistent with prior published work and were in clinically elevated ranges, including inhibit, shift, working memory, plan/organization, and monitor (White et al., 2017). Adolescents with AN had scores higher than population norms on the shift, initiate, and emotional control scales (as well as the BRI and GEC); however, these scores were not above the clinical cutoff. This is consistent with prior work indicating that adolescents with AN scored, on average, in the normal range but had elevated scores compared to healthy controls on the shift scale of the BRIEF (McAnarney et al., 2011).

Adolescents with AN demonstrated clinically elevated scores on the inhibit, shift, monitor, and emotional control scales compared to healthy controls (Figure 1). The inhibit scale is associated with impulse control, the shift scale is associated with flexibility, and the monitor scale assesses one’s awareness of the impact of their behavior on others and awareness of staying on task. The emotional control scale assesses how EF impacts an adolescent’s ability to regulate both positive and negative emotions in relation to caregivers and peers. Adolescents with AN had average scores well below the clinical cutoff on all scales. Only one female with AN had elevated scores on the inhibit scale, 22.7% (*N* = 5) had elevated scores on shift, and 13.6% (*N* = 3) had elevated scores on monitor. An examination of other scales indicated that the adolescents in the AN sample had elevated scores on emotional control, although not in the clinical range (Figure 1). Elevated scores on this measure indicate that caregivers reported that their child has emotional outbursts, mood lability, and periods of emotional upset. Adolescents with eating disorders will often become upset around meal times, may refuse to eat, and can become aggressive. It is possible that elevated scores on this scale are state-based and a result of behavior linked directly to restricted eating. This scale is also one of the only ones where adolescents with AN were more similar to adolescents with ASD. Further research is needed to see if this is a state-based difference or a true endophenotype.

In order to gain more insight into how females with AN were rated compared to females with ASD, we examined the percentage of females who met the clinical cutoffs on each scale. As highlighted in Figure 2, the majority of scales had over 50% of adolescents with ASD in the clinical range; the only two scales with less than 50% of adolescents with ASD in the clinical range were emotional control (43.3%) and organization of materials (46.7%). In comparison, there was a maximum of 31.8% of females with AN who reached the clinical cutoffs on a total of three scales: emotional control, initiate, and organization of materials.

Previous studies in ASD have established a link between shift impairments and restricted and repetitive behaviors. However, those samples are predominantly male, and so it was not known if this relationship would hold in an all-female ASD sample, given that one study showed this relationship to be male specific (Bölte et al., 2011). We saw a similar significant correlation in our sample between shift and RRBs as has been found in predominantly male samples. Given the emerging evidence that females on the autism spectrum may present as unique from males, (Parish-Morris et al., 2017) it is critical for the field and clinicians to understand where the ASD phenotype overlaps and where it is unique in males and females. Furthermore, considering sex-specific profiles of ASD could lead to better diagnostic recognition of ASD in females and enhance clinical practice to support autistic females.

These results have several implications for future research. Given the lack of clinically significant set-shifting inefficiencies observed in the female sample with AN in this study, it is unclear if set-shifting inefficiency is truly an endophenotype of AN or if it is a consequence of extended periods of malnutrition (as in adult samples). Short-term fasting can result in a measurable decrease in set-shifting ability, (Benau et al., 2014) so it is very possible that a prolonged period of malnutrition could result in routines and rules that occur around food becoming ingrained. It is also possible that malnutrition derails EF development and that the inefficiencies observed in set-shifting in adults with AN are a scar of the illness. We still cannot rule out that a sub-group of adolescents has difficulties in set-shifting and that these individuals are at risk for a longer course of illness. If so, it is likely that the latter group represents the adults who are captured in published studies that support the narrative of a shared endophenotype of set-shifting difficulties. Regardless, it is clear that future work into the neuropsychological underpinnings of similar behavioral topology in AN and ASD needs to occur in adolescent samples who are followed longitudinally. Future work needs to involve sex-matched controls to test for differences in pubertal hormone influx and their impact on EF development. It is also important that future work consider how other risk factors – such as genetic vulnerabilities, temperament (e.g., harm avoidance), psychiatric co-morbidities, or environmental factors may impact EF. Finally, a heterogeneous sample is necessary in order to reflect the variation of psychiatric symptoms typical in AN and ASD populations.

## Conclusion and Limitations

This study provides a first glimpse into understanding the overlapping and distinct features of the EF profile in female adolescents with ASD or AN diagnosis. Caregivers of adolescent females with AN did not consistently rate them in the clinical range of difficulties in EF. They were also not rated similarly to adolescent females with ASD. While the sample is small, this study significantly adds to the literature exploring the overlap between ASD and AN. Future work should include performance-based measures of EF as well as self-report (when possible) and caregiver report. These complimentary measures will provide a more comprehensive picture of inefficiencies in set-shifting and EF more broadly. While there is no consistent data on the rates of ASD in AN samples, work in adult samples indicates that it could be as high as 22.9% (Huke et al., 2013). Future work exploring the co-morbidities needs to continue in adolescents.

There were several limitations to this study. The exclusion of males minimizes our ability to comment on the similarity of the EF profiles of male ASD and AN adolescents. The female youths included in this analysis were only identified for their diagnosis of restrictive eating or ASD, without consideration for other neurodevelopmental or psychiatric diagnoses, particularly attention deficit/hyperactivity disorder (ADHD). Previous studies have indicated that co-occurring ADHD affects EF in youth with ASD, (Wallace et al., 2016; Yerys, 2020) and depression that is often co-morbid with anorexia can impact EF. We did not have information on body weight/height for the youth with ASD; thus, we were not able to control for this variable. As this was a secondary data analysis, we did not have consistency in the assessment of IQ; however, IQ measures tend to be highly correlated with one another. As we collapsed across measures for the ASD sample, we doubt that this had an impact on the results. Prospective research will allow for the standardized assessment of IQ across both groups. It is possible that there are group differences in the caregivers that we were not able to control for in this secondary data analysis which could have driven the differences in EF observed. Future work should characterize the caregivers in order to explore this possibility. We also focused on neuro-cognitive functions; future work in adolescent samples should include measures of EF, social cognition, and response to social reward. Finally, the sample size was small, and we conducted a number of comparisons; these results must be replicated in larger, fully powered, prospective samples.

## Data Availability Statement

The data analyzed in this study is subject to the following licenses/restrictions: Data is available upon request to the corresponding author. Requests to access these datasets should be directed to catimko@pennmedicine.upenn.edu.

## Ethics Statement

The studies involving human participants were reviewed and approved by Institutional Review Board, Children’s Hospital of Philadelphia. Written informed consent to participate in this study was provided by the participants’ legal guardian/next of kin.

## Author Contributions

CAT: conceptualization, methodology, formal analysis, writing – original draft, and resources. JH and EK: writing – review and editing and resources. AB: data curation, formal analysis, and writing – original draft. BY: conceptualization, methodology, writing – review and editing, and resources. All authors contributed to the article and approved the submitted version.

## Conflict of Interest

The authors declare that the research was conducted in the absence of any commercial or financial relationships that could be construed as a potential conflict of interest.

## Footnotes

1 The data presented here are either unpublished or a small (<10%) subsample of published data sets. Information on other publications is available from the author upon request.
